# Annual Research Review: Exposure to environmental chemicals and psychosocial stress and the development of children's learning difficulties

**DOI:** 10.1111/jcpp.14137

**Published:** 2025-03-18

**Authors:** Amy E. Margolis, Alex Dranovsky, David Pagliaccio, Gazi Azad, Virginia Rauh, Julie Herbstman

**Affiliations:** ^1^ Department of Psychiatry and Behavioral Health, Wexner Medical Center The Ohio State University Columbus OH USA; ^2^ Child Mind Institute New York NY USA; ^3^ Division of Systems Neuroscience, Department of Psychiatry, College of Physicians and Surgeons Columbia University New York NY USA; ^4^ Heilbrunn Department of Population and Family Health, Mailman School of Public Health Columbia University New York NY USA; ^5^ Department of Environmental Health Sciences, Mailman School of Public Health Columbia University New York NY USA

**Keywords:** Learning difficulties, environmental exposures, brain development, stress

## Abstract

Although awareness of the role of environmental exposures in children's cognitive development is increasing, *learning difficulties* have not yet been a major focus of environmental health science. Learning difficulties disproportionately affect children living in economic disadvantage, yielding an ‘achievement gap.’ Studies examining the neurobiology of reading and math have mostly included economically advantaged youth, leaving a great deal unknown about the neural underpinnings of reading and math difficulties in youth living in disadvantaged contexts. Critically, due to environmental injustice, these youth are disproportionately exposed to environmental neurotoxicants. Herein, we review literature supporting a theoretical framework of *environmentally associated phenotypes of learning difficulties*. We propose that prenatal exposure to neurotoxicants *and* early‐life exposure to psychosocial stressors increases risk for learning difficulties via effects on neural circuits that support cognitive processes which, in addition to literacy and numeracy, are integral to acquiring and performing academic skills. We describe models in which (1) prenatal exposure to air pollution has a main effect on learning via brain structure and function or associated domain‐general cognitive processes and (2) a joint ‘two‐hit’ pathway in which prenatal air pollution exposure followed by early life stress—when combined and sequential—increases risk for learning difficulties also via effects on brain structure, function, and/or associated cognitive processes. We review a select literature documenting effects of exposure to pollutants and early life stress on relevant neural circuits and associated cognitive processes in animal models and parallel findings in human epidemiologic studies. We advocate for team science in which researchers, practitioners, and policymakers collaborate to increase health literacy about *environmentally associated phenotypes of learning difficulties* and support the development of precision‐oriented instructional and environmental intervention methods for youth living in economic disadvantage.

## Introduction

In the United States, youth living in economically disadvantaged contexts have disproportionately more difficulty acquiring reading and math skills than do their advantaged peers (Garcia, 2015; Miller, Votruba‐Drzal, & Coley, 2019). In fact, 50%–70% of youth living in economic disadvantage have serious reading or math problems (EdTrust, 2024; National Literacy, 2024). This disparity—commonly referred to as the ‘achievement gap’—has significant personal and societal costs including increased rates of mental health problems, school dropout, unemployment, substance use, and incarceration (Aro et al., 2019; Fakier & Wild, 2011; Francis, Caruana, Hudson, & McArthur, 2019; Gerber, 2012; Rimrodt & Lipkin, 2011; The Lancet Public Health, 2021; Williamson, 1992).

The underlying etiology of these reading and math problems varies as a function of socioeconomic status (SES). Twin studies have shown that among children living in higher socioeconomic status (SES) contexts, genetic factors largely explain the variance in word reading skills and intelligence, whereas the environment accounts for most of the variance in lower SES contexts (Friend, DeFries, & Olson, 2008; Haughbrook, Hart, Schatschneider, & Taylor, 2017; Peterson & Pennington, 2015; Shero et al., 2024; Taylor & Schatschneider, 2010). But notably, some studies have not reported similar results for intelligence or reading comprehension (Bates, Hansell, Martin, & Wright, 2016; Little, Haughbrook, & Hart, 2017). Critically, environmental contributors to learning difficulties examined in prior studies include household or personal environmental factors, such as parent education and its passive influence on the home environment and/or parent's active involvement with child's engagement in stimulating activities, as well as a child's likelihood of reading, which decreases among those with reading problems (Peterson & Pennington, 2015). Furthermore, having more adverse childhood experiences (ACES) has been linked with having poorer academic outcomes (Bethell, Newacheck, Hawes, & Halfon, 2014). However, exposure to neurotoxic chemicals in the environment, such as ambient air pollutants, has been overlooked as a potential contributor to learning difficulties (Wodtke & Parbst, 2017). These chemical exposures also disproportionately affect youth living in the context of economic disadvantage and are collectively considered issues of environmental justice (*Environmental justice primer for ports: Defining environmental justice*, 2019). Air pollution, for example, is disproportionately higher in lower versus higher income neighborhoods based on systematic placement of pollution sources near these neighborhoods and oppressive structural factors that keep people residing in these neighborhoods (Hajat, Hsia, & O'Neill, 2015; Us Epa O, 2019). Moreover, the effects of exposure to air pollution may be magnified by or may magnify the effects of social stressors (Margolis, Liu, et al., 2022), which also affect neural function and are more prevalent among children living in the context of economic disadvantage (Evans & Kim, 2013). These neurotoxic environmental exposures are particularly deleterious during the prenatal and early life periods, when the brain is undergoing rapid growth and, thus, is most vulnerable (Grandjean & Landrigan, 2006).

Strong evidence from non‐human, experimental animal models provides causal evidence of the harmful effects of prenatal exposures to a range of common and neurotoxic chemicals on brain development and subsequent behavior (Grandjean & Landrigan, 2006). Researchers and clinicians now appreciate that developmental neurotoxicants may be important but overlooked etiologic factors in neurodevelopmental disorders (Grandjean & Landrigan, 2006). Perhaps, most well known in humans are causal links between lead exposure, brain changes, and reduced intellectual ability (Lanphear et al., 2005). The etiologic role of neurotoxicants in reading and math difficulties, however, remains relatively understudied (Margolis, Greenwood, Dranovsky, & Rauh, 2023; Rauh & Margolis, 2016). In our work, we have established links between a number of different neurotoxic chemicals (e.g. prenatal exposure to air pollution, second hand smoke, flame retardant chemicals) and changes in brain structure and function, as well as downstream effects on cognition and academic achievement (Cowell et al., 2018; Margolis, Banker, et al., 2020; Margolis, Pagliaccio, et al., 2021). Critically, these exposures rarely occur in isolation, leading the field to consider effects of multiple exposures, or ‘mixtures’ of exposure.

The compounding effects of multiple exposures, including the interaction between neurotoxic chemicals and psychosocial stressors on mental health, have become a focus in environmental health science research in the past decade (Padula et al., 2020). In our work, we have developed a theoretical framework of *environmentally associated phenotypes of learning difficulties* (Margolis et al., 2023) in which prenatal exposure to neurotoxic chemicals such as air pollution are associated with problems in cognition, learning, and academic skills. We also hypothesize a ‘two‐hit’ model in which prenatal exposure to air pollution followed by early life stress, when *combined and sequential*, has independent and joint effects on academic outcomes. Supporting this theoretical framework, the chapter on Specific Learning Disorder in the Diagnostic and Statistical Manual‐5‐TR (DSM‐5‐TR) (American Psychiatric Association, 2022) now recognizes chemical and social exposures as risk factors, and some researchers have hypothesized environmental chemicals as potential underlying causes of specific learning disorders (Wodtke & Parbst, 2017). Nevertheless, we lack a clear understanding of the neurobiological and cognitive mechanisms underlying these phenotypes. In this focused review, we present relevant literature to support this framework and identify possible neural and cognitive mechanisms that may underlie two such *environmentally associated learning difficulties* phenotypes. We then propose interdisciplinary approaches that can move the field of learning difficulties research forward by illuminating possible targets for future educational, behavioral, and pharmacologic intervention and for public health prevention.

This review is aimed at enhancing our etiologic understanding and practical remediation of learning difficulties by viewing it through the lens of inequity and environmental injustice. We provide support for inclusive research and training that will engage in translational science for the benefit of practitioners and policymakers and for youth who reside in socially and economically disadvantaged communities. The review covers three major themes: (1) characterizing *environmentally associated phenotypes of learning difficulties* that contribute to disadvantage‐related educational disparities and exemplify environmental injustice; (2) understanding etiological and neurobiological mechanisms underlying learning difficulties’ phenotypes through the lens of interdisciplinary, translational neuroscience, and public health; and (3) encouraging targeted engagement of learning difficulties researchers, practitioners, and policymakers in a collaborative process of shared learning using the principles of implementation science to translate research findings into real‐world changes in public policy and the instruction of youth with learning difficulties.

**Figure 1:**
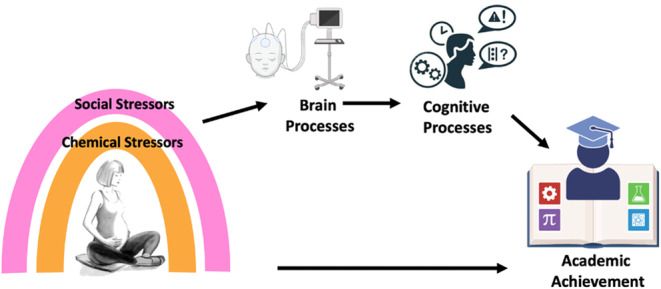
Conceptual diagram of proposed model

We first define the construct of learning difficulties and discuss reasons for considering learning difficulties within a continuous, skill‐level framework (e.g., based on performance on standardized tests) rather than a categorical, diagnostic framework. Second, we review the literature on the neurobiological underpinnings of academic skills and learning difficulties and consider potential brain‐dependent mechanisms that could underlie exposure‐related learning phenotypes. Third, we review the evidence showing links between prenatal exposure to air pollution, psychosocial stressors, and performance on tests of academic skills, and discuss data analytic approaches to the inherent problem of studying mixtures of correlated exposures. Fourth, we review evidence from human epidemiologic and animal models showing the effects of air pollutants and psychosocial stressors on the brain and associated cognitive processes. Ultimately, we present evidence of two *environmentally associated phenotypes of learning difficulties* describing (1) links between prenatal exposure to air pollution and learning difficulties via reduced hippocampal volumes and inhibitory control and (2) links between combined and sequential exposure to prenatal air pollution followed by early life stress and learning difficulties via reduced hippocampal volume, altered cingulo‐opercular function and lowered attention capacity (see Figure 1 for a conceptual overview). Last, we discuss future directions for how community‐engaged research can help move the field forward towards more inclusive and effective research and practice.

## Identifying and defining learning difficulties

Quantifying and labeling learning problems in youth from economically disadvantaged backgrounds is problematic, from both phenomenological and measurement standpoints. Learning problems are labeled in the psychology/psychiatry clinic as specific learning disorder, but in the educational setting are given a classification (not diagnosis) of learning disability. Further complicating the issue is the Americans with Disabilities Act, which specifies when a disorder rises to the level of a disability. Rigorous prior studies show that inconsistency in applying the criteria for the diagnosis of *specific learning disorder* and/or educational classification of *learning disability* leads to both over‐ and under‐identification of learning difficulties, defined herein as performance in the bottom quartile on academic tasks (Morgan, Farkas, Hillemeier, & Maczuga, 2017; Shifrer, Muller, & Callahan, 2011). Shifrer et al. reported that disproportionate over‐identification of African American and Hispanic students with *learning disability* is largely explained by lower average SES of these racial/ethnic groups, and that identification of *learning disability* is associated with sex, socio‐demographic characteristics, and academic history, as well as being a language minority learner (Shifrer et al., 2011). In contrast, Morgan et al. repeatedly found that ‘minority children are less likely to be identified as having disabilities’ and, as a consequence, receive fewer special education services than ‘otherwise similar white children’ across separate nationally representative data sets. They further reported that under‐identification attributable to race or ethnicity as well as to sex, English language learner status, and ‘low income’ background status has been occurring in the US since at least 2003 (Morgan et al., 2017).

Based on these inconsistencies in identifying *learning disability* in children from ethnic/racial minoritized backgrounds and economically disadvantaged families—groups often underrepresented in research—we focus on skill level, defining *learning difficulties* as performance in the bottom quartile of standardized reading or math tests. This approach also aligns with ‘response to intervention’ models (Otaiba, Kim, Wanzek, Petscher, & Wagner, 2014), which identify children at risk for learning problems based on lower skills performance rather than a diagnosis.

## The neural signature of learning difficulties in disadvantaged youth remains poorly understood

**Figure 2:**
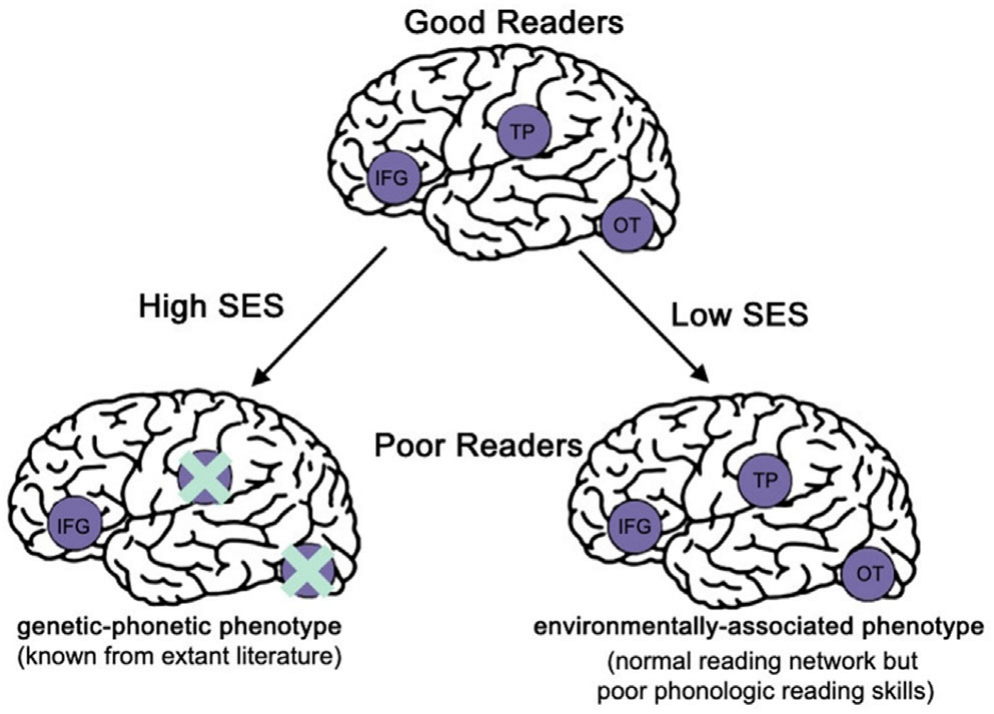
Conceptual figure representing the left‐lateralized reading network function and dysfunction in typical and atypical higher versus lower SES readers. IFG, inferior frontal gyrus; OT, occipitotemporal cortex; SES, socioeconomic status; TP, temporoparietal cortex

Critically, children living in the context of economic disadvantage have been largely underrepresented in prior magnetic resonance imaging (MRI) studies investigating the neurobiology of reading and math ability and difficulty. For nearly two decades, these MRI studies have established the neural correlates of domain‐specific reading processes, such as phonological processing, as tied to a left‐lateralized ‘reading network’ in adults and children who are good readers (Martin, Schurz, Kronbichler, & Richlan, 2015). Furthermore, studies have shown that this left‐lateralized ‘reading network’ is under‐engaged in poor readers during reading or phonological processing tasks (Martin et al., 2015). These findings have collectively come to be understood as defining the ‘neural signature of dyslexia.’ But these studies have largely relied on individuals from higher SES, advantaged backgrounds. Emerging evidence suggests that the neurobiology of learning difficulties varies with SES (Conant, Liebenthal, Desai, & Binder, 2017; Gullick, Demir‐Lira, & Booth, 2016; Noble, Wolmetz, Ochs, Farah, & McCandliss, 2006; Ozernov‐Palchik et al., 2019; Romeo et al., 2022; Van der Mosten, Boets, Wouters, & Ghesquière, 2012; Younger, Lee, Demir‐Lira, & Booth, 2019) and cannot fully be accounted for by lack of adequate instruction (Wodtke & Parbst, 2017). A seminal paper showed that, among poor readers from lower SES backgrounds, the canonical left‐lateralized ‘reading network’ is engaged during reading‐related phonological tasks, despite youth having poor phonological skills (Figure 2) (Noble et al., 2006). Furthermore, we have recently shown that volumes of cortical regions in the left‐lateralized ‘reading network’ are not associated with word reading skills among youth living in disadvantaged contexts, further dispelling the idea that the neural signature of dyslexia is universal across all youth (Marcelle et al., 2024). Additionally, one other recent study showed an interaction between the neural signature of reading problems and SES such that, among students from higher SES backgrounds, neural differences between typical readers and those formally diagnosed with learning disabilities are most prominent in the frontal portions of the reading network during phonological tasks, whereas differences between good and poor readers from lower SES backgrounds are most prominent in posterior regions of the reading network (Romeo et al., 2022). In sum, these data suggest that the neural correlates of reading problems in youth from economically disadvantaged backgrounds are distinct from those reported in youth from higher SES, advantaged backgrounds.

The neurobiology of math is also narrowly understood, similar to the extant literature on the neurobiology of reading. Studies of the neurobiology of math learning disability diagnosis identify dysfunctional engagement and connectivity of the intraparietal sulcus that is associated with poor working memory (Menon & Chang, 2021) and more recently, the critical role of the hippocampus in math learning disability diagnosis (Menon & Chang, 2021). Historically, studies have not focused explicitly on understanding the neural circuits underlying math in youth living in disadvantaged contexts nor have they examined effects of SES on the neural underpinnings of math learning difficulties. New findings, however, suggest that SES moderates the neural correlates of math skills (Demir, Prado, & Booth, 2015; Demir‐Lira, Prado, & Booth, 2016; Johnson, Burgoyne, Mix, Young, & Levine, 2022). These findings underscore the importance of considering SES in the neurobiology of math and suggest that, as is the case for reading skill, the neural correlates of math difficulties in youth from economically disadvantaged backgrounds are distinct from those reported in youth from higher SES, advantaged backgrounds.

We propose that the neurobiological pathways that contribute to learning difficulties among children from economically disadvantaged families seem to differ from those of other children. Specifically, we hypothesize that learning difficulties in children living in economic disadvantage arise from alterations in domain general circuits which support general cognitive processes that contribute to reading and math skills. This difference is in place of, or in addition to, alterations in domain‐specific circuits such as the left‐lateralized reading circuit which underlies phonological processes in advantaged youth. These pathophysiologic differences in learning difficulties are critical to understand because they may be reflected by different treatment needs and responses in the two populations. However, these differences remain understudied (Miller, Vaughn, & Freund, 2014), hindering the development of new interventions to improve academic achievement among youth living in economically disadvantaged contexts. One plausible place to search for such alternate pathways is in cognitive processes that preferentially enhance academic performance in these youth, and to examine if (1) the function of brain regions and circuits that support these processes explains variance in academic skills performance or risk for learning difficulties, and (2) if these regions, circuits and processes are vulnerable to environmental (chemical or social) exposures. In what follows, we take this approach to delineate a pathway to proposed *environmentally associated phenotypes of learning difficulties*.

## Neurocognitive processes that support learning and academic skills

As mentioned above, models of the neurobiology of reading and math have largely focused on neural processes tied to domain‐specific processes, such as phonological awareness or numeracy, that explain reading and math difficulties, respectively. In the past two decades, however, there has been an explosion of knowledge in the field of systems neuroscience. This work identifies how the brain processes information and makes computations that underlie *learning processes*. The role of these domain‐general learning systems in the acquisition of academic skills remains understudied. In our theoretical model, we build on foundational systems neuroscience literature to hypothesize that acquisition of math and reading skills depend on such domain‐general capacities. From a neural standpoint, we argue that the cortical and subcortical circuits which underlie domain‐general processes likely explain variance in reading, either alongside or instead of domain‐specific processes. Notably, subcortical circuits have been particularly overlooked in MRI studies examining the neurobiology of reading and math. Here, we focus specifically on two domain general processes—working memory and inhibitory control—and their associated neural circuitry, as they have been identified as critical to reading and reading problems (Marcelle et al., 2024; Margolis, Pagliaccio, et al., 2020). We acknowledge that there are other domain‐general processes, such as complex attention, that are important for reading (Goldman, 2024) but which have not yet been studied as mediators in models of environmental exposures. We propose that working memory and inhibitory control are not functioning as expected in our model of environmentally associated phenotypes of learning difficulties. We further hypothesize that this disfunction explains more variance in learning difficulties in youth living in economic disadvantage than in their advantaged peers. We focus on the hippocampus and dopamine system as potential candidates, as they have been associated with both working memory and inhibitory control and are targets of environmental exposures under study (see below).

### Working memory and learning difficulties

Working memory, defined as the ability to hold information in mind and simultaneously manipulate that information, is involved in reading and math in both typically developing students and those with learning disabilities or learning difficulties (Daucourt, Schatschneider, Connor, Al Otaiba, & Hart, 2018; Nouwens, Groen, & Verhoeven, 2017; Schurer, Opitz, & Schubert, 2020; Slattery, Ryan, Fortune, & McAvinue, 2021). Examples include keeping text in mind and problem‐solving when making an inference, or completing mid‐procedure calculations as required during long division. Few studies have examined the role of working memory in reading or math problems in youth living in the context of economic disadvantage (Diuk, Barreyro, Ferroni, Mena, & Serrano, 2019). Critically, however, youth living in the context of disadvantage are at risk for impaired working memory, and this lower level of functioning is mediated by exposure to chronic stress (Evans & Schamberg, 2009). We hypothesize that exposure to chemicals and social stressors that act as neurotoxicants target working memory and have downstream effects on reading and math for these youth.

The hippocampus is well known for its significant role in long‐term declarative memory (Squire & Zola, 1996). Recent work also highlights an important role for the hippocampus in working memory and to dorsolateral prefrontal cortex (Dimakopoulos, Mégevand, Stieglitz, Imbach, & Sarnthein, 2022; Li et al., 2022). Specifically, in presurgical epilepsy patients, performance during a working memory task varied with hippocampal function (Li et al., 2022), and the encoding and maintenance of information during working memory appears to be driven by activity between the auditory cortex and hippocampus (Dimakopoulos et al., 2022). Recent evidence also implicates the hippocampus in reading comprehension (Aboud, Bailey, Petrill, & Cutting, 2016; Hausser et al., 2021) and in math (Mathieu et al., 2018; Menon, 2016). However, neuroimaging studies have not specifically interrogated the role of the hippocampus in academic skill performance in general or among youth with reading or math learning difficulties in particular. Based on robust evidence linking working memory with reading and math (Daucourt et al., 2018; Menon, 2016; Nouwens et al., 2017; Schurer et al., 2020; Slattery et al., 2021), it is possible that working memory may mediate associations between the hippocampus and academic skills.

### Inhibitory control and learning difficulties

Inhibitory control includes the ability to inhibit an automatic, prepotent response and instead instantiate a less automatic response—for example, saying ‘go’ when a red light appears, or ‘up’ to name a downward facing arrow. Children's early self‐regulation capacities, including inhibitory control, have been linked with reading and math skills (McClelland & Cameron, 2011). This association has been observed specifically in children from economically disadvantaged backgrounds (Finders, McClelland, Geldhof, Rothwell, & Hatfield, 2021). Specifically, lower inhibitory control has repeatedly been linked with poor reading and math outcomes (McClelland & Wanless, 2012). Additionally, training inhibitory control in preschool improves math outcomes (McClelland et al., 2019), suggesting a causal path between improving inhibitory control and math skills (McClelland et al., 2019). Inhibitory control appears to play a critical role in children's academic skill acquisition not only through its effect on staying on task but also through its role in executing academic processes. First, individuals with reading disorder often show deficits in cognitive control processes, although these studies were not conducted in populations living in economic disadvantage (Booth, Boyle, & Kelly, 2010; Mahé, Doignon‐Camus, Dufour, & Bonnefond, 2014; Reiter, Tucha, & Lange, 2005; Varvara, Varuzza, Sorrentino, Vicari, & Menghini, 2014). Second, inhibitory control processes associate with reading skills, including word reading, reading fluency, and reading comprehension (Kibby, Lee, & Dyer, 2014). Finally, computational models of latent processes underlying inhibitory control errors show a reduced drift rate during motion processing (Manning et al., 2021) and during visual and auditory lexical decision tasks, indicating difficulty extracting and processing information in children with reading disorders (Manning et al., 2021; Zeguers et al., 2011).

Inhibitory control is governed by cognitive control neural circuits. Different names for these control circuits and variations on regions included in them can be found in the literature, but the general consensus is that inhibitory control involves cingulo‐opercular and frontoparietal brain regions. These systems and inhibitory control behaviors are modulated by dopamine signaling, as is well documented in animal and human studies (Ott & Nieder, 2019). Specifically, this involves dopamine (Ott & Nieder, 2019) projections to the associative striatum and prefrontal cortex.

Cognitive control circuits and inhibitory control processes may play an important role in the learning difficulties phenotype. Prior findings from our group and others suggest that individuals with reading disorder show altered activation of control circuits during control tasks as well as altered functional connectivity of task control circuits (Horowitz‐Kraus, Hershey, Kay, & DiFrancesco, 2019; Horowitz‐Kraus, Toro‐Serey, & DiFrancesco, 2015; Koyama et al., 2013; Margolis, Pagliaccio, et al., 2020; Schurz et al., 2015). As these circuits and processes are implicated in reading disorder in higher SES populations, and inhibitory control problems are disproportionately observed in children from disadvantaged backgrounds, these inhibitory control circuits and processes may be preferentially involved in academic skill performance among youth who are living in economically disadvantaged backgrounds.

## Economic disadvantage differentially exposes children to chemicals and psychosocial stressors

Resolving health disparities has become a recent focus for policy and scientific advancement. These disparities arise in part through environmental injustice, defined as the systemic oppression of minoritized groups through disproportionate exposures to toxicants. Environmental injustice leads to children living in the context of economic disadvantage having the greatest exposure to neurotoxic chemicals. These children are disproportionately exposed to chemicals such as air pollutants due to greater placement of exposure sources near low‐income, urban communities (Hajat et al., 2015). Critically, these communities are also disproportionately composed of Black and Latinx individuals (Hajat et al., 2015). Furthermore, structural factors such as living in homes with poor ventilation may lead to ongoing exposure. Additionally, youth living in the context of disadvantaged backgrounds also tend to experience disproportionate exposure to psychosocial stressors (Evans & Kim, 2013).

## Defining and measuring exposures

Here, we describe strengths and limitations of approaches to measuring exposure to air pollution and psychosocial stressors, as well as biostatistical models for handling mixtures of exposure.

### Air pollutants

Complexity in measuring exposure to chemical pollutants arises from the many types of pollutants that can be measured and from different measurement methods. For example, the different ways of characterizing air pollutants include: (1) source (e.g., traffic‐related air pollutants (TRAP), including black carbon, nitrogen oxides, tire dust); (2) size (e.g., particulate matter (PM) typically classified by whether they are smaller than 2.5 or 10 μm in diameter); and (3) composition (e.g., polycyclic aromatic hydrocarbons (PAHs), with hundreds of known neurotoxic congeners including benzo[a]pyrene, chrysene, benzo[b]fluoranthene). Critically, these pollutants are produced by both indoor and outdoor sources, and thus, different measurement methods will capture exposure from different sources. Air pollutants can be measured via area‐level monitoring (e.g., with data from regional stationary monitoring stations and/or computer modeling using combinations of station data, satellite images, and meteorological variables) or via personal monitoring (e.g., personal air samplers and/or biomarkers). These approaches can be complementary and vary in their cost and resolution, but also have unique strengths and weaknesses which can lead to exposure misclassification.

Personal monitoring using active or passive devices will capture an individual's indoor and outdoor exposure as they move throughout their daily lives. Although high spatial resolution is achieved with portable personal air monitors, these monitors are typically deployed for only a limited time to reduce participant burden or to limit cost. Active samplers use filters that collect or measure particles or vapor from the air for a set period of time, capturing diurnal and seasonal alterations in pollutants. Passive monitors are also used, which absorb pollutants into a porous matrix (e.g., silicone). Biomarkers enable personal estimation of exposure but are subject to the half‐lives of the analytic targets in biological samples and require the collection of biological samples. Again, these measures are highly accurate with respect to exposure at the time of collection; however, this could reflect an unusual circumstance for an individual. Highly time‐resolved personal biomarker assessment could pose significant and unreasonable burden on participants, making it an unlikely avenue for assessment in most circumstances.

In contrast to personal monitoring of exposure, many studies leverage area‐level monitoring to estimate exposure for geographic regions or individual addresses (for a contemporary review, see Holloway 2021 (https://ntrs.nasa.gov/api/citations/20230000905/downloads/Holloway_review_satellite)). Satellite data yield high temporal resolution for exposure measurement and can even be calculated at an hourly interval. Critically, this approach is limited to measuring outdoor exposure (i.e., cannot capture sources within an individual's home), although a significant portion of indoor exposure is related to conditions outside. Furthermore, this approach assumes individuals spend equivalent and consistent amounts of time at their address. Nonetheless, models for several different exposures have been made available to researchers and can be easily linked via geocodes to specific data collected in a variety of different contexts by participant address (e.g., linking to large‐scale cohort studies or smaller well‐characterized samples). Furthermore, this approach allows researchers to link pollution estimates with other regional metrics like greenspace or noise pollution.

### Psychosocial stressors

Psychosocial stressors occur at the individual, household, and neighborhood level. As in measuring air pollution, these psychosocial stressors can be measured at the individual level with different metrics. Most commonly, assessment will include individual self‐report surveys or interviews about experiences of stressful events, including acute events and/or chronic stressors (Hellhammer, Stone, Hellhammer, & Broderick, 2010). Self‐report of stressors is a key way to assess individual experiences but is limited by intrinsic biases of all self‐reports (e.g., the limits of retrospective recall, discomfort in disclosing of trauma) (Lalande & Bonanno, 2011). When studying stress exposure in children, many studies will employ parental report, which again is powerful but subject to these same limitations and issues in disclosing family trauma or abuse and work, showing that a parent with their own stress exposure or mental health concerns may be more likely to report more stressors for their child (Gauthier‐Légaré, Tarabulsy, Ouellet, Gagné, & Langlois, 2022). One commonly used measure is the adverse childhood experiences (ACES) survey, which is typically administered retrospectively (Felitti et al., 2019). In our longitudinal prospective cohorts, we have used repeated administration of survey measures to examine distinct types of stressors including maternal perceived stress, maternal psychological distress, material hardship, neighborhood quality, intimate partner violence, and maternal intimate partner violence. Additionally, population‐based measures have been developed to geocode adversity at the neighborhood level via composite scores (e.g., the Area Deprivation Index (ADI) or social vulnerability index (SVI)). The ADI ranks neighborhoods by socioeconomic disadvantage (income, education, employment, and housing quality) and can be coded to the census block (Neighborhood Atlas – Home). The SVI is often used in environmental justice research to parse social determinants of health (SDOH) measures that contribute to health outcomes. The SVI captures relative vulnerability in each census tract based on 15 indicators across four themes (socioeconomic status; household characteristics; racial and ethnic minority status; housing type and transportation). Other work includes assays of hormones (e.g., cortisol, or inflammatory markers to measure the biological impact of stress) (Marsland, Walsh, Lockwood, & John‐Henderson, 2017). These methods typically are leveraged to assess either reactivity of the stress system to an acute in‐lab stressor (Goodman, Janson, & Wolf, 2017) or prolonged accumulation of stress exposure (Hellhammer et al., 2010).

### Mixtures of exposure

Analyzing and interpreting chemical exposure data, as described above, is complicated because chemical exposures frequently co‐occur, posing unique challenges to biostatistical modeling. Many studies have examined the effect of a single exposure, which can provide critical insights into effects of pollution but may not be mechanistically specific to a given compound or account for interactions or confounding between exposures. Several statistical approaches have been developed and adapted to investigate the effects of multiple exposures to probe different questions (Gibson et al., 2019). For example, one may aim to identify which chemical among a set of co‐occurring chemicals is the deleterious component or ‘bad actor.’ This issue is commonly addressed via methods like weighted quantile sum (WQS) regression. WQS is strong in its ability to apportion variance to highly correlated predictors within a single model but is weak with respect to analyzing direction of effects. Bayesian kernel machine regression (BKMR), in contrast, can show direction of effects and can assess statistical interactions between exposures but does not isolate single bad actors. In addition, understanding patterns in the co‐occurrence of exposures is a separate question commonly addressed with clustering techniques. These approaches can identify patterns in exposure source, type, or dose. For example, principal components pursuit (PCP) transforms a high‐dimensional exposure matrix into a low‐rank matrix, which identifies consistent patterns, and a sparse matrix, identifying unique events that cannot be explained by the common patterns (Gibson et al., 2022; Tao et al., 2023). In an exposome approach, the totality of an individual's exposures are measured simultaneously to establish combined effects of co‐exposures and identify which chemicals are negatively influencing an outcome of interest (The exposome, 2020).

Critically, in addition to co‐exposure to chemicals, a burgeoning literature points to the importance of considering the co‐occurrence of exposure to chemicals, such as air pollutants, and psychosocial stressors (Padula et al., 2020), which co‐occur in part because both are associated with structural factors that disproportionately affect economically disadvantaged individuals (Evans & Kim, 2013; Hajat et al., 2015). Considering the joint effects of air pollution and early life stress on cognition and learning, as we propose here, may help capture the dynamics at play in these learning difficulty phenotypes. Underscoring this approach, our group has documented a profile of high exposure to air pollutants (including PAH and environmental tobacco smoke) and psychosocial stress (material hardship, maternal demoralization) among disadvantaged youth in our cohorts, providing evidence for an air pollutant and stress exposure phenotype (Benavides, Kourmotgalou, Herbstman, Pagliaccio, & Margolis, n.d.).

## Exposures increase risk for learning difficulties

Below we review studies that have linked environmental exposures, specifically air pollution and early life stress, with cognitive ability and/or academic outcomes.

### Air pollutants

Higher prenatal and early‐life exposure to air pollution is associated with worse academic skills (Grineski, Collins, & Adkins, 2020; Lett, Stingone, & Claudio, 2017; Stingone, McVeigh, & Claudio, 2016, 2017). Specifically, in a study of 16,000 children, high exposure to a mixture of toxic air pollutants in kindergarten predicted lower reading, math, and science scores in third grade (Grineski et al., 2020). Moreover, in 201,559 urban children, high exposure to a mixture of toxic air pollution at birth was associated with low math scores and increased likelihood of receiving academic support services in third grade (Stingone et al., 2016, 2017). Preschool math scores were also predicted by exposure to isophorone, an ambient air marker of industrial air pollution, in infancy (*N* = 4,050) (Lett et al., 2017). Although these prior studies include large samples, they rely on area‐level models to estimate exposure based on census tract, rather than measuring personal exposure, likely contributing to *under*‐estimated effect sizes due to exposure misclassification.

Using personal exposure measurements, our group has documented links between lowered academic skills and higher prenatal exposure to PAH (Margolis, Ramphal, et al., 2021; Yang et al., 2025). PAHs are neurotoxic carcinogens produced during incomplete combustion of fossil fuels, tobacco, and other organic material (Boström et al., 2002). Epidemiologic evidence suggests that *prenatal* PAH exposure has deleterious effects on child health and development (Grandjean & Landrigan, 2006; Volk et al., 2021). In one quasi‐experimental study, the closure of a coal‐burning plant leads to lower biomarker of PAH exposure in newborns, upregulation of protective brain‐derived neurotrophic factor (BDNF) protein, and better neurocognitive development at 2 years of age (Tang et al., 2014). In our studies, we have linked prenatal PAH with lowered reading comprehension and math skills, via pollution‐related effects on inhibitory control (Margolis, Ramphal, et al., 2021), and with lowered word reading via effects on hippocampal volumes (Yang et al., 2025).

### Psychosocial stressors

Large epidemiologic studies document effects of psychosocial stressors on learning difficulties. In a study of 95,677 children from across the United States, those with two or more adverse childhood experiences (ACEs) were 2.67 times more likely to repeat a grade in school versus those with no ACEs who were 2.59 times more likely to be engaged in school, as defined in the National Survey of Children's Health (Bethell et al., 2014). Poverty was the strongest predictor of suspension and poor academic outcomes in a study of 181,897 Florida students (Balfanz, Byrnes, & Fox, 2014).

Finally, chemical and social stressors interact to affect learning difficulties. In prospective longitudinal birth cohort studies, our group has shown that prenatal PAH exposure increases vulnerability to the effects of early life stress (ELS) on neural, cognitive, behavioral, and academic outcomes in youth (Greenwood et al., 2023; Margolis, Cohen, et al., 2022; Pagliaccio et al., 2020; Vishnevetsky et al., 2015). First, children with higher prenatal PAH and more ELS have reduced hippocampal subfield volumes, which were associated with lower performance on visual–spatial tests (Margolis, Cohen, et al., 2022). Additionally, a separate cohort showed that higher prenatal PAH and material hardship were associated with lower IQ at 7 years of age (Vishnevetsky et al., 2015), and greater attention and thought problems at four time points from age 3 to 11 years of age (Pagliaccio et al., 2020). Finally, youth with higher prenatal exposure to PAH and material hardship showed lower reading scores (Greenwood et al., 2023).

### Summary

Air pollutants and psychosocial stressors, both independently and jointly, have been associated with reductions in academic skill performance and learning difficulties. However, the biological processes underlying these associations are poorly understood. In what follows, we consider how brain‐dependent mechanisms may mediate these exposure‐related phenotypes.

## Animal and human evidence linking exposures with neural processes

Evidence from animal models and human epidemiologic studies links environmental exposures to altered neural structure and function, as well as to disrupted neurotransmitter signaling processes. Animal models have notable strengths in that exposure is well quantified and can be causally manipulated to disentangle the independent effects of exposures that frequently co‐occur in the human condition, such as air pollution and psychosocial stress. As we have noted previously, only a handful of studies have examined whole brain effects of exposure to air pollution; studies are needed to examine effects of exposure across the whole brain rather than testing hypothesis‐driven regions or processes (Margolis, Liu, et al., 2022).

Importantly, although academic skills per se cannot be studied in animal models, learning processes can. A robust animal model literature documents causal effects of exposure to air pollutants and psychosocial stressors on brain and behavior processes. Human epidemiologic studies show associations between these exposures, neural structure and function, and cognitive processing. As reviewed above, these domain‐general cognitive processes have been linked to academic skills allowing inference for how exposures may ultimately affect academic skill performance, especially in disadvantaged populations. In what follows, we align findings from animal models of exposure that show causal effects on domain‐general circuits and human epidemiologic studies showing associations between exposure and neural processes. We elect to focus on two neural processes—the hippocampus and the dopamine transmitter system—as they have both been shown to be targets of air pollution and stress. However, we acknowledge that there are likely other neural regions and systems that are targets of pollution and stress and may be involved in the proposed models.

### Hippocampus and air pollutants

In animals, prenatal ambient PM2.5 exposure causes reductions in hippocampus volume (Klocke et al., 2017), altered hippocampus morphology including decreases in the number of dendritic branches and terminals in hippocampal subregions CA1 and CA3 (Tseng et al., 2019), disrupted neural integrity in the hippocampus (Nephew et al., 2020), and reduced neurogenesis in the dentate gyrus (Woodward et al., 2018). Furthermore, pre‐ and postnatal TRAP exposure reduced BDNF expression in the hippocampus and cortex of mice (Liu et al., 2019; Miller et al., 2016; Zhou et al., 2020). Behavioral findings parallel these anatomical findings. Early life exposure to PAH is associated with impaired learning and memory in a T‐maze test as measured by decreased time required to reach a reservoir between initial and final trials in zebrafish (Gao et al., 2017). In mice, prenatal exposure to PM2.5 reduces working memory (Tseng et al., 2019) as measured by more spontaneous alternation errors in a cross maze (Kulas et al., 2018) and causes spatial memory problems such as prolonged latency to find the platform and decreased platform crossing times in the Morris water maze (Tseng et al., 2019; Zhou et al., 2020). Notably, these effects persist into third‐generation female offspring who were not prenatally exposed to PM2.5 (Zhou et al., 2020). Additionally, prenatal PAH exposure reduced novelty preference, likely affecting learning (Miller et al., 2016).

Consistent with these findings, studies in humans document associations between prenatal exposure to air pollutants and altered hippocampal structure in youth between 7 and 14 years old, some drawn from lower SES (Margolis, Cohen, et al., 2022; Peterson et al., 2022), and some from higher SES backgrounds (Lubczyńska et al., 2021). Paralleling the finding in animal models that pollutant exposure reduced hippocampal BDNF (Liu et al., 2019; Miller et al., 2016; Zhou et al., 2020), closing of a coal burning plant resulted in increased BDNF expression in the human umbilical cord and improved developmental outcomes (as described above) (Tang et al., 2014). In humans, prenatal exposure to several air pollutants (PM2.5, black carbon, residential roadway proximity) have been linked with decreased working memory, visual–spatial memory, and problem‐solving in youth between 6 and 14 years old, with some studies including youth from lower SES (Chiu et al., 2013, 2016; Suglia, Gryparis, Wright, Schwartz, & Wright, 2008) or from higher SES backgrounds (Harris et al., 2015; Rivas et al., 2019).

### Hippocampus and psychosocial stress

Experimental exposure to stress impairs hippocampal structure and function in animal studies (Fenoglio, Brunson, & Baram, 2006; Margolis, Liu, et al., 2022; Rocha, Wang, Avila‐Quintero, Bloch, & Kaffman, 2021). Multiple reports have documented how experimental exposure to early life stress impairs hippocampus structure and function (Walker et al., 2017). For example, mice exposed to chronic stress (including early life stress) have decreased hippocampus volume (Walker et al., 2017). Furthermore, mice exposed to early life stress exhibit delays in dentate gyrus development and lifelong changes in hippocampal stem cell function (Youssef et al., 2019). Psychological stress (e.g., restraint stress, housing in dominance hierarchies, maternal deprivation) also results in suppression of neurogenesis, loss of spine synapses in hippocampal subregions, and shortened dendrites (McEwen & Gianaros, 2010). Chronic restraint stress results in neuronal atrophy in CA3 and CA1 subfields of the hippocampus (McEwen, 1999; Watanabe, Gould, & McEwen, 1992). Other forms of stress, either chronic or acute, decrease neurogenesis in the dentate gyrus (McEwen, 1999; McEwen & Gianaros, 2010). In addition, these stress interventions impair long‐term potentiation and synaptic plasticity in both CA1 and dentate gyrus subfields (Alfarez, Joëls, & Krugers, 2003). Changes in cortisol and glutamate, both of which have been linked to hippocampal structure and function and are associated with the stress response, could mediate neuronal remodeling (McEwen & Gianaros, 2010). These changes could underlie the morphological findings commonly observed in human stress research (Kassem et al., 2013).

In humans, exposure to stress during early life is a known risk factor for lifelong cognitive and emotional impairment (Pechtel & Pizzagalli, 2011). Human brain MRI studies have repeatedly found that stress is associated with reduced hippocampus volume (Barch & Pagliaccio, 2020). This association has been one of the most replicated findings in human MRI research (Bromis, Calem, Reinders, Williams, & Kempton, 2018; Calem, Bromis, McGuire, Morgan, & Kempton, 2017; Woon, Sood, & Hedges, 2010). Studies also implicate the effects of broader, stress‐related constructs, such as SES, in relation to hippocampus volume deficits (Brito & Noble, 2014; Luby et al., 2013). We and others have reported associations between early life stress and altered hippocampal structure and function (Pagliaccio et al., 2014, 2015). Numerous studies have identified links between early life stress and cognitive impairments in adulthood and in youth, including working memory deficits (Goodman, Freeman, & Chalmers, 2019; Op den Kelder, Van den Akker, Geurts, Lindauer, & Overbeek, 2018). This association has been found across a variety of working memory tasks, including phonological and visuospatial tasks, as well as in individuals with and without psychopathology (Goodman et al., 2019).

### Combined exposures differentially impact hippocampus

In mice, prenatal exposure to a combination of diesel exhaust particles and maternal stress impairs learning and social interaction and disrupts network connectivity of a frontal circuit that includes the hippocampus (Block et al., 2022; Bolton et al., 2013; Smith et al., 2022). Importantly, neither learning nor social impairments were observed following exposure to either diesel exhaust or stress alone, supporting the idea that perinatal stress compounds the effects of exposure to environmental toxicants.

In humans, we recently reported that prenatal PAH exposure moderated the association between maternal perceived stress at child age 5 and right hippocampus CA1, CA3, and CA4/dentate gyrus subfields volumes at child age 7–9 years (*n* = 37) (Margolis, Cohen, et al., 2022). Specifically, higher prenatal PAH exposure magnified negative associations between maternal stress and child hippocampus volume, whereas this relation was buffered at lower PAH exposure. Performance IQ was significantly associated with right hippocampus CA3 and CA4/dentate gyrus subfield volumes (*B* > 0.35, *t* > 2.16, *p* < .04).

In sum, human epidemiologic studies and animal models show that independent exposure to prenatal PAH or ELS is related to hippocampal structure and function, as well as dependent cognitive processes, including working memory. *Combined* exposure to prenatal PAH and early life stress has been associated with hippocampal structure, attention, and word reading in children and adolescents living in disadvantaged contexts (Greenwood et al., 2023; Margolis, Cohen, et al., 2022; Pagliaccio et al., 2020) and in animal models combined exposure impairs learning and disrupts network connectivity of frontal circuits with the hippocampus (Block et al., 2022; Bolton et al., 2013; Smith et al., 2022). We propose that the timing and degree of exposure to pollutants and stressors likely results in alterations to hippocampal structure and function, which in turn gives rise altered cognitive processing, in this case working memory, and ultimately underlies a learning difficulties phenotype.

### Dopamine and pollutants

In animal models, prenatal exposure to air pollution alters dopamine and its metabolites across the prefrontal cortex and the dorsal and ventral striatum, key regions in cognitive control processes. Prenatal exposure to the PAH benzo‐[a]‐pyrene reduces dopamine level and induces dopaminergic neuron loss across the zebrafish brain (Gao et al., 2017). Studies of prenatal exposures to other air pollutants also show decreased dorsal striatal dopamine turnover (Suzuki et al., 2010; Yokota et al., 2009), alterations in the ventral striatum (Yokota et al., 2009; Yokota, Oshio, Moriya, & Takeda, 2016), and increased levels of dopamine in the prefrontal cortex (Yokota et al., 2016). To our knowledge, only one rodent model has examined the ventral tegmental area (VTA), a source of dopamine, and produced null findings (Yokota et al., 2016). In these rodent models, dopaminergic‐mediated behavioral change has also been observed after prenatal exposure to pollutants (Margolis, Liu, et al., 2022). For example, TRAP exposure increase aggression via effects on prefrontal cortical dopamine (Yokota et al., 2016).

Consistent with these findings from experimental models, PAH exposure in humans is linked to changes in brain structures associated with dopamine function. For example, exposure to PAH has been linked with reduced caudate volumes in school‐age children (Mortamais et al., 2017). In addition, prenatal exposure to particulate matter is associated with reduced cortical volume in infants. In addition to looking at dopamine‐served structures in the brain, neuromelanin scanning is a validated, novel method for estimating dopamine function that is safe for use in children (Cassidy et al., 2019; Wengler, He, Abi‐Dargham, & Horga, 2020).

### Dopamine and stressors

Although acute stress has been linked with increased dopamine firing in the mesolimbic and mesocortical dopamine system, chronic stress can yield decreased dopamine in the VTA and nucleus accumbens (NAc), sources of dopamine production (Baik, 2020; Holly & Miczek, 2016). Critically different chronic stressors (e.g., food deprivation vs. chronic restraint) have different neurobiological outcomes (Baik, 2020), underscoring the importance of examining granular measures of early life stress. Furthermore, animal models show links between exposure to early life stress and acute stress and altered reward processing, which is governed by dopamine (Carvalheiro, Conceição, Mesquita, & Seara‐Cardoso, 2021; Duque‐Quintero et al., 2022; Hanson, Williams, Bangasser, & Peña, 2021).

Based on their small size, the VTA and NAc have been difficult to isolate in human MRI research. Advances in MRI technology have increased spatial resolution, allowing examination of the VTA in humans for the first time. Recent work has begun to implicate ELS in altered VTA connectivity, for example, with medial prefrontal cortex [PFC] and HC (Marusak, Hatfield, Thomason, & Rabinak, 2017; Park et al., 2021), and reduced reward‐related functional MRI activity in the VTA (Richter, Krämer, Diekhof, & Gruber, 2019).

## Hypothesized phenotypes

**Figure 3:**
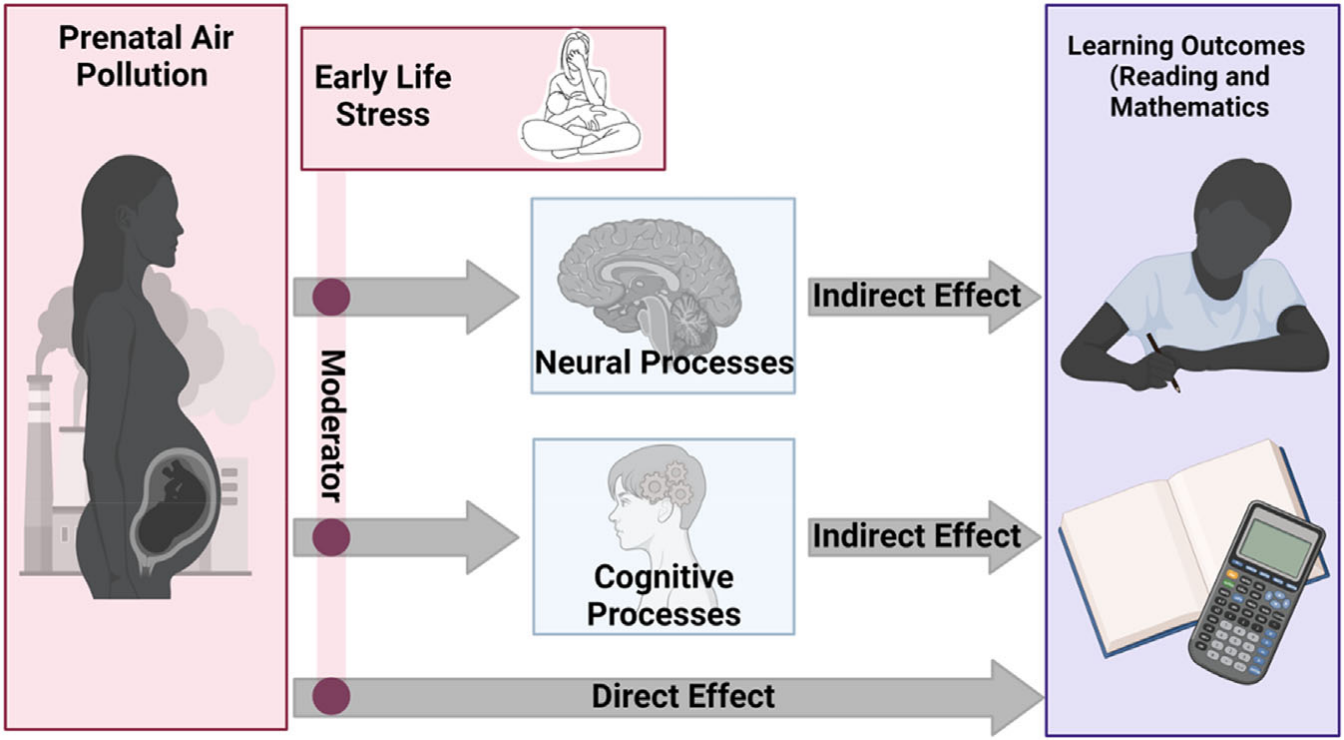
Diagram of the models for the proposed phenotypes

Aligned with this review of the literature, we have developed a research program aimed at identifying neurobiological and cognitive mechanisms that contribute to these environmentally associated phenotypes of learning difficulties (Figure 3). Here, we review two avenues of inquiry that have been most productive thus far. These studies have been conducted in the longitudinal birth cohort studies conducted at the Columbia Center for Children's Environmental Health. Participants in these cohort studies reside mainly in Northern Manhattan and Washington Heights, report relatively low household income and maternal education. More than 90% of the cohort self‐identify as Black and/or Latine. Results are summarized in Table 1. In future planned studies, we will examine these models in larger national cohorts such as the Environmental influences on Children's Health Outcomes (ECHO) Program (ECHO, 2020).

**Table 1:** Examples of data supporting the two hypothesized phenotypes

	Exposure	Neurobiological outcomes	Cognitive outcomes	Academic outcomes
Hypothesized Phenotype 1: Main Effect of Chemical Exposure	PAH	Reduced hippocampal CA2/3 subfield volume (Yang et al., 2025)	Slower change over time in parent‐rated self‐regulatory capacity on the CBCL (Margolis et al., 2016) Worse NEPSY Inhibition subtest performance (Margolis, Ramphal, et al., 2021)	Lower reading comprehension (Margolis, Ramphal, et al., 2021) Lower word reading (Margolis, Ramphal, et al., 2021) Lower basic math (Margolis, Ramphal, et al., 2021)
Hypothesized Phenotype 2: Joint Effect (Two‐hit) of Chemical and Psychosocial Exposures	PAH + material hardship	Reduced hippocampal subfield volumes (Margolis, Cohen, et al., 2022)		Reduced pseudoword reading (Greenwood et al., 2023)
	SHS + postnatal maternal distress	Increased global efficiency of the cingulo-opercular network (Greenwood et al., 2024)	Higher efficiency of the cingulo‐opercular network associates with attention problems and ADHD risk (Greenwood et al., 2024)	

Abbreviations: ADHD, attention deficit hyperactivity disorder; CA, cornu ammonis; CBCL, Child Behavior Checklist; ELS, early‐life stress; PAH, polycyclic aromatic hydrocarbon; SHS, second‐hand smoke.

### Air pollutants, inhibitory control, and reading and math skills

Our hypothesized pathway from PAH to academic skill via inhibitory control mechanisms rests in part on prior findings that inhibitory control is important for academic skill acquisition, as reviewed above, and also on findings that PAH exposure has been associated with self‐regulatory processes. We have previously shown that prenatal exposure to PAH is associated with the delayed development of self‐regulatory capacities, as rated by parents on the Child Behavior Checklist (CBCL) (Margolis et al., 2016). In that work, prenatal exposure interacted with self‐regulation over time; we observed slower change in self‐regulatory capacity over time in exposed children (*p* = .05). In a follow‐up study, we examined if PAH‐related effects on self‐regulation were associated with adolescent academic skills. We estimated prenatal exposure to PAH using personal monitoring of exposure. During the third trimester of pregnancy, mothers wore an air monitoring backpack for 48 continuous hours. We used children's direct performance on a measure of inhibitory control rather than parent report of self‐regulatory behavior. We measured inhibitory control with the NEPSY Inhibition subtest at mean age = 10.4 years and academic skills with the Woodcock‐Johnson Tests of Achievement‐III (WJ‐III) at mean age = 13.7 (*N* = 200 participants). We found that higher prenatal PAH exposure was associated with worse child inhibitory control, worse spelling skills, and worse reading comprehension and math skills (at trend level). Reading comprehension and math skills were positively associated with inhibitory control. Finally, a mediation analysis showed that inhibitory control mediated associations between prenatal PAH exposure and passage comprehension (*β* = −0.61, 95% CI: −1.49, −0.01) as well as a Broad Math Index (*β* = −1.09, 95% CI: −2.36, −0.03) (Margolis, Ramphal, et al., 2021).

We are currently investigating neural mechanisms underlying this cognitive pathway. In recent work, we documented associations between prenatal PAH DNA‐adducts and adolescent word reading skills that are mediated by hippocampal volumes (Yang et al., 2025). Based on the possible role of hippocampus in inhibitory control processes (Hu & Li, 2020), we hypothesize that altered hippocampal volume may be part of the underlying pathway in this phenotype. Using task fMRI, we are examining if neural activation in cognitive control circuits during a control task mediates these PAH‐related effects on reading and math, across a wide range of ages. We are also using neuromelanin‐MRI to examine this question and investigate the role of dopamine in these pathways.

### Air pollutants, psychosocial stressors, and reading in young children

Based on findings that psychosocial stress co‐occurs with air pollution exposure and is associated with both reduced inhibitory control and academic skill acquisition, we hypothesized that exposure to *both* psychosocial stress and PAH might have joint effects on academic outcomes. To test this ‘double‐hit’ hypothesis, we examined the combined effects of prenatal PAH exposure followed by different aspects of economic disadvantage on learning difficulties (Greenwood et al., 2023). We tested if exposure to any of eight material hardships at child age 5 (living conditions, food, housing, billing, clothing, health‐care, Medicaid access, and public assistance) interacted with prenatal PAH exposure, measured via personal air monitoring. Academic skills were measured using the WJ‐III Basic Reading Index at child age 7. Analyses controlled for ethnicity/race, sex, birthweight, presence of a smoker in the home during pregnancy (a proxy for secondhand smoke (SHS) exposure), and maternal education. The prenatal PAH by material hardship interaction was associated with WJ‐III Basic Reading Index (*β* = −.347, *t* (44)= − 2.197, *p* = .033), and in follow‐up analyses, this effect appeared to be driven by an untimed measure of pseudoword reading (WJ‐III Word Attack: *β* = −.391, *t* (44)= − 2.550, *p* = .014) (Greenwood et al., 2023).

We also tested the timing of the ‘double hit’ exposure in another recent pilot study. In this small sample (*N* = 32), we examined if *combined and sequential* exposure to prenatal second hand smoke (SHS)—which contains PAHs—followed by early childhood exposure to psychosocial stress altered the function of cognitive control circuits at child age 7–9 (Greenwood et al., 2024). We used graph theoretical methods to test if the global efficiency of the frontoparietal or cingulo‐opercular task‐positive networks was associated with these early life exposures. The prenatal SHS by postnatal maternal distress interaction term was significantly associated with the global efficiency of the cingulo‐opercular network (*p* = .02) but not the frontoparietal network *(ns)*. Higher efficiency of the network was also associated with more attention problems and ADHD risk (*p*'s < .01). Critically, no significant main effects of prenatal SHS or postnatal maternal distress on network efficiency were observed. Furthermore, the interaction between prenatal SHS exposure and postnatal maternal distress with network efficiency remained significant when postnatal SHS or prenatal maternal distress were included in the model (*p* = .03, *p* = .03, respectively), pointing to the importance of the timing of exposure. Although this small pilot study did not include academic skills as an endpoint, it does lend initial support to the double‐hit hypothesis and may point to an environmentally associated pathway to academic challenges that rest on underlying deficits in executive processes such as inhibitory control.

## Future directions

Our research program has four areas for growth that we think are critically important for the field.

### Biomarkers of learning difficulties

The neural and molecular bases of learning difficulties can potentially serve as biomarkers of risk and diagnosis, as well as therapeutic targets and treatment response. As noted throughout, both PAH and ELS independently have been linked to downstream pathways to learning difficulties, but also may create a ‘double hit’ via shared mechanistic pathways. A complex understanding of the neurobiology of the two‐hit exposure (pollution followed by stress) pathway could provide multiple entry points for improving child outcomes. We are far from understanding this process that likely has many complex mediating pathways. Alterations in brain structure/function and inflammation represent possible shared mechanistic pathways. For example, PAH has been linked to widespread alterations in cortical structure and downstream associations with ADHD outcomes (Peterson et al., 2015) and effects of perinatal stress on ADHD outcomes have been linked to cortical maturation (Bock & Braun, 2011). We have ventured to hypothesize that one way in which this ‘two‐hit’ pathway could operate is via BDNF. Some evidence suggests this could be a plausible path for further inquiry. In humans, serum BDNF is positively associated with stress exposure. Concurrently, BDNF is reduced by exposure to air pollutants, specifically PAHs (Margolis, Liu, et al., 2022). Thus, prenatal exposure to PAH could reduce available BDNF which in turn could lower resilience to stress in early childhood. Animal models point to hippocampal BDNF being particularly vulnerable to PAH (Margolis, Liu, et al., 2022) and stress exposures (Radecki, Brown, Martinez, & Teyler, 2005; Taliaz et al., 2011). Coupled with the well‐replicated finding that stress is negatively associated with hippocampal volume in humans, it is reasonable to posit that sequential exposure to first PAH and then stress could underlie such morphometric changes via effects on hippocampal BDNF. Indeed, in our group, we have shown that combined and sequential exposure to PAH followed by stress is associated with reduced hippocampal volumes, albeit in a relatively small sample. Understanding the mechanisms of this two‐hit pathway can allow entry points for intervention and prevention. Critically, BDNF is but one growth factor which may be operating in this model; we advocate for an ‘omics’ approach to understanding growth factors and hormone function in these models.

### Novel academic intervention programs

With great success, the science of reading has focused a great deal of energy on the importance of phonological processing for reading. We now advocate for more complex approaches that incorporate what has been learned in systems neuroscience and treatment of other neuropsychiatric disorders (e.g., pediatric anxiety) to the treatment of learning problems. For example, reading problems are well known to be associated with working memory problems, but not all youth with reading problems have working memory deficits. If we can more deeply characterize reading problems and develop reading interventions that incorporate strategies for improving working memory *in the context of word reading*, then we may move closer to precision education techniques. One similar example can be found in work addressing anxiety within the context of reading instruction. Anxiety symptoms and reading problems are known to be associated and posited to exist in a bidirectional relationship. Vaughn and colleagues have developed specific reading interventions that incorporate cognitive behavioral techniques for reducing anxiety for use in mainstream classrooms. This program leads to both reductions in anxiety symptoms as well as increases in reading comprehension in typically developing readers. Future work is aimed at testing these approaches for youth with reading deficits where effect sizes may be larger. Critically, there is scant research on environmentally associated phenotypes of learning difficulties and no public health initiatives to translate and disseminate this information to promote health literacy in the community. However, increasing the communities' awareness about environmentally associated phenotypes of learning difficulties may lead to more precision‐oriented instructional methods (e.g., those that limit the focus on working memory) for disadvantaged youth.

### Community engagement

More than two decades ago, seminal educational work documented that it takes up to 17 years for just 14% of research findings to benefit real‐world practice settings such as public schools (Balas & Boren, 2000). This research‐to‐practice lag spawned the transdisciplinary field of implementation science, defined as the study of methods to promote the uptake of evidence‐based practices into real‐world service delivery with the goal of optimizing clinical outcomes and shifting public policy (Rapport et al., 2018). A foundational construct in implementation science is that research should be conducted in partnership *with*, and not *for*, the relevant communities to achieve lasting public health impact.

Community engagement requires working collaboratively with others based on geographical proximity, attributes or special interests to address common issues or concerns and is a complex iterative process (Blachman‐Demner, Wiley, & Chambers, 2017; CTSA Community Engagement Key Function Committee Task Force, 2011). In the learning difficulties field, agents of change may operate in distinct educational communities including researchers (basic science, clinical and epidemiologic), practitioners (educators, learning disability specialists and parents who interact with local schools), and policymakers (administrators, representatives and government officials)—all of which comprise the wider educational system. Engaging the wider educational community will be essential for the translation of research findings to the practice and policy communities, where they can potentially result in measurable improvements in individual treatments for learning difficulties and, at the population level, reduce inequities in educational opportunities for at risk children living with social and environmental disadvantage.

We have identified four critical needs in the learning difficulties field: (a) enhanced systems for training a professional learning difficulties workforce with the transdisciplinary skills required to work in collaborative teams across research, practice, and policy settings (Kirchner, Smith, Powell, Waltz, & Proctor, 2020); (b) dedicated pathways to independence for early‐career scientists and practitioners from underrepresented groups (Oh et al., 2015); (c) effective dissemination of new learning difficulties research findings to the wider educational practice and policy communities for potential testing, implementation, and scaling (Sanetti & Luh, 2019); and (d) feedback systems linking the wider educational communities (practice and policy/governance) back to the community of academic researchers to share information about their lived experiences, unmet needs, and lessons learned across diverse settings.

**Figure 4:**
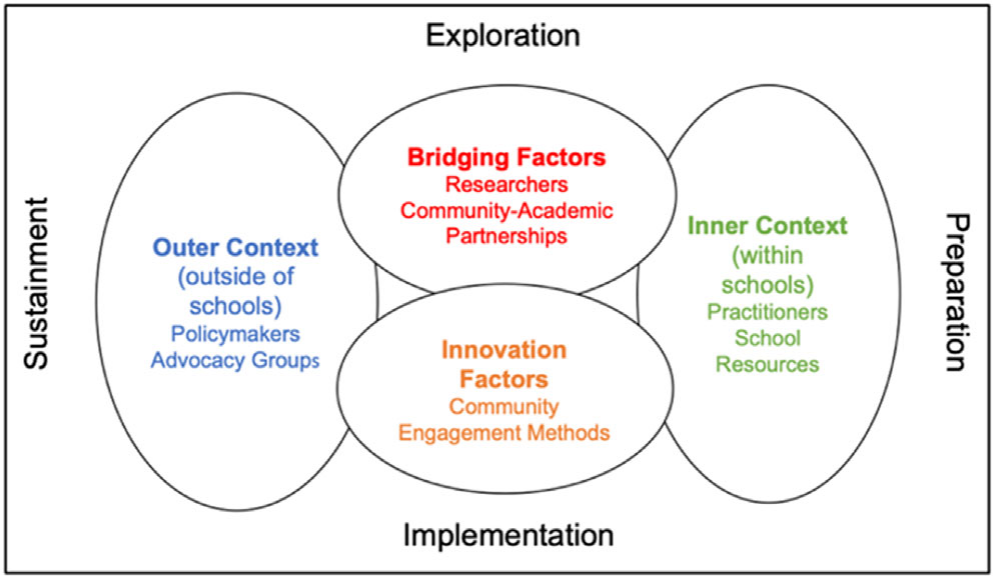
Key components of the EPIS framework

The EPIS framework describes the key phases of the implementation process (Exploration, Preparation, Implementation, and Sustainment) (Moullin, Dickson, Stadnick, Rabin, & Aarons, 2019) and has been used to support community‐partnered research (Figure 4). EPIS considers factors in the outer system context (e.g., policy), inner organizational context (e.g., educators), and those that bridge contexts (e.g., community–academic partnerships), as well as characteristics of the innovation (e.g., teaching methods for learning difficulties). Although EPIS has been used for translational science in medicine, social science, and public health, its use in the field of education is limited. There is a critical need to translate advances in treating learning difficulties into practice and policy to insure equitable application of new innovations for disadvantaged students, who are often at greatest risk of learning difficulties, yet this work is scant (Miller et al., 2014). We propose that future community engagement and implementation efforts could adopt the EPIS framework to address gaps in learning difficulties research described above. For example, EPIS may be used to guide the development of a community‐driven health literacy program about environmentally associated phenotypes of learning difficulties. During the initial *Exploration* phase, it would be important to consider the emergent or existing needs of the community. For example, it would be important to consider the extent of knowledge about prenatal environmental exposures, early life stressors, and learning difficulties. In the *Preparation* stage, barriers and facilitators would be discussed, and a plan would be developed to leverage facilitators and minimize barriers. During the *Implementation* stage, it would be important to implement the community‐driven health literacy intervention at a few vanguard sites. Finally, during the sustainment stage, it would be critical to follow those who receive the health literacy training to determine the extent to which it impacted their instructional approaches with students with learning difficulties (e.g., less reliance on working memory).

### Public health prevention programs

We have described learning difficulties as a costly public health problem and one that disproportionately affects children living in the context of social and economic disadvantage. From a public health perspective, the inequity in levels of exposure to both environmental pollutants and social stress constitutes a form of ‘environmental injustice’—a condition in which some people and communities are less protected than others from environmental and health hazards, including those related to structural or systemic barriers. The growing public health problem of widespread exposure in the general population to a range of potentially neurotoxic chemicals is a relatively recent source of concern to the medical and public health communities, especially with respect to potential early brain compromise in children (Rauh & Margolis, 2016). This development argues for effective regulatory activity, but the high volume of new chemicals being introduced annually and the need for updated safety standards have challenged the capacity of the United States. EPA to manage the risk assessment process.

Ultimately, the prevention of exposure‐associated learning difficulties is a complex systems‐wide problem that will require the engagement of neuroscientists, educators, healthcare practitioners, government officials, and policymakers—each group with its own language, training programs, and regulatory standards. As noted above, principles from implementation science can be used to engage these different communities to come together to shift best practices and public health policies, including regulatory action. In light of long‐standing achievement gaps and embedded structural inequities, the challenge for the public health system is daunting and will require truly interdisciplinary team science at it best.

## Summary, conclusions, and future directions

*Environmentally associated* phenotypes of reading and math difficulties disproportionately affect disadvantaged youth (Evans, 2004; Garcia, 2015; Hajat et al., 2015; Miller et al., 2019), but this burden has not yet been a major focus of environmental health science. We propose that prenatal exposure to air pollution and early life stress are overlooked determinants of health in the etiology of these learning difficulties. Despite being over‐represented among those with learning difficulties, disadvantaged youth have historically been underrepresented in neuroimaging studies of reading and math (Miller et al., 2014). Critically, alterations in the left‐lateralized ‘reading network’ that underlie reading problems in advantaged youth do not explain reading problems in disadvantaged youth (Marcelle et al., 2024; Noble et al., 2006). As a result, current learning difficulties interventions which are largely based on neural and cognitive processes identified in studies of majority, advantaged populations may not be generalizable or even effective for disadvantaged youth. To close the ‘achievement gap,’ precision‐oriented instructional interventions must address the effects of environmental chemical and social exposures in order to improve reading and math skills in disadvantaged youth.

We advocate for a translational approach to understanding links between environmental exposures (e.g., air pollutants and psychosocial stressors), and neural (e.g., hippocampal structure, inhibitory control circuits, or dopamine signaling among many others) and cognitive (e.g., working memory and inhibitory control among others) processes. Animal models allow experimental manipulation of exposure and a shorter timescale for observing outcomes. For example, cognitive and behavioral effects of gestational PM_2.5_ exposure persisted into *third‐generation female offspring who were not prenatally exposed* (Zhou et al., 2020). Such findings point to the importance of reducing exposures and understanding the intergenerational transmission of effects of exposures via epigenetic and other mechanisms. Animal models can also identify tools for reversing the cognitive and behavioral consequences of exposure. For example, chemogenetic activation of dopamine neurons restored sociability in the offspring of air pollutant and stress‐exposed dams, suggesting that the dopamine system may be a viable target for therapeutic intervention following these prenatal exposures (Block et al., 2022). These kinds of experiments hold promise to identify treatment targets for learning difficulties resulting from environmental exposures.

As has been suggested throughout this review, human studies can also be designed to try and understand mechanisms of exposure‐related outcomes. Twin studies examining differential stress exposures and learning outcomes could employ MRI to help establish causal mechanisms underlying the pathway from environmental (chemical and social) exposures to learning difficulties. Innovative fMRI tasks can identify dysfunction in circuits that are distinct from domain‐specific circuits associated with reading and reading problems (or math and math problems) and that govern domain‐general learning processes of working memory and inhibitory control. Using computational models to parse specific subcomponents of behavior beyond typical performance metrics may provide more nuanced targets for personalized interventions. As has been shown for example, in children with reading disorder, impaired accumulation of knowledge appears to underlie poor performance on conflict monitoring tasks (Manning et al., 2021; Zeguers et al., 2011). This kind of detailed knowledge can serve as the basis for developing precision‐oriented instructional intervention activities or as markers of treatment efficacy.

Critically, we also advocate for a community‐engagement component of research that can translate finding from the laboratory to the classroom. Working within an implementation science framework, results from translational studies can serve as the groundwork for developing novel, community‐informed precision‐oriented instructional and environmental interventions. Principles from implementation science can help translate causal evidence from animal models to the human condition and into real‐world practice, ultimately optimizing clinical outcomes and shifting public policy. A foundational construct in this rapidly growing field is that research should be conducted in partnership *with*, and not *for*, the relevant communities to yield lasting public health impact. Specifically, for example, novel precision‐oriented instructional tools for learning difficulties might incorporate training for working memory directly into reading instruction. This interdisciplinary approach, bringing together researchers/trainees, practitioners, and policymakers from the fields of education, neuroscience, and public health, promises new solutions to address serious, persistent yet unacceptable learning gaps for vulnerable children.

**Key points**

- Learning difficulties disproportionately affect disadvantaged youth and increase risk of mental health problems, school dropout, unemployment, substance use, and incarceration.
- Studies examining the neurobiology of reading and math have largely included demographically homogenous and economically advantaged individuals.
- The neural underpinnings of learning difficulties in youth living in economically disadvantaged contexts are largely unknown.
- Due to environmental injustice, youth living in economically disadvantaged contexts are disproportionately exposed to neurotoxicants.
- We hypothesize *environmentally‐associated phenotypes of learning difficulties* that derive from exposure to environmental neurotoxicants and alter neural circuits and cognitive processes integral to academic skills.
- Pathophysiologic differences in learning difficulties between advantaged and disadvantaged youth require precision‐oriented instructional and environmental interventions.

## Data Availability

No original data are presented in this manuscript.
